# Supplementary material for: Long-Term Effects of Microfiltered Seawater and Resistance Training with Elastic Bands on Hepatic Parameters, Inflammation, Oxidative Stress, and Blood Pressure of Older Women: A 32-Week, Double-Blinded, Randomized, Placebo-Controlled Trial
Source: Healthcare (Basel). 2024 Jan 15;12(2):204. doi: 10.3390/healthcare12020204 (PMC10815454; doi:10.3390/healthcare12020204)
Supplement: Supplementary file 1 [file healthcare-12-00204-s001.zip › healthcare-2757623-supplementary.pdf]

PRESENT 2020 (PROPER REPORTING OF EVIDENCE IN SPORT AND EXERCISE NUTRITION TRIALS) CHECKLIST [a]

Section	Item	Checklist	Page/Line Number (if applicable)
**Title**			
	1a	State the independent (groups/conditions) and dependent (outcome) variables	Page 0, lines 1–2
	1b	Identify the study population or case	Page 0, lines 3–4
**Abstract**			
	2a	Specify the research design, methods, and characteristics of the study population	Page 0, lines 11–13
	2b	Report a balanced account of the results and cite actual data	Page 0, lines 13–20
	2c	Restrict conclusions to measured variables, without speculation or unsupported recommendations	Page 0, lines 20–23
**Introduction**			
	3a	Present a scientific rationale based on an objective review of available evidence	Pages 1–2, lines 27–63
	3b	State the aims, objectives, research questions, and/or hypotheses	Pages 2–3, lines 64–74
**Methods**			
Ethics	4	Provide details of ethical approval (citing conduct of human research in accordance with the Declaration of Helsinki)	Page 3, lines 80–86
Design	5	Summarize the research design (e.g., parallel trial/cross-over, randomized, counterbalanced, blinding, or observational)	Page 3, lines 78–80
Sampling	6a	List the eligibility (inclusion/exclusion) criteria and sampling method	Page 4, lines 96–102, 112–114
	6b	Characterize the study sample (e.g., demographics, anthropometry, and lifestyle)	Page 9, lines 208–217, Table 1
	6c	Report the setting/location and periods of recruitment and data collection	Acknowledgements Section
	6d	Justify the sample size (presenting the selected target effect size and error variances to replicate sample size estimates)	Pages 7–8, lines 184–188
Interventions [b]	7	Detail all aspects of the groups/conditions (considering the need to verify the composition of ingested substances)	Pages 5–6, lines 117–148
Measurements	8a	Define the pre-specified primary, secondary and/or mechanistic outcome variables	Pages 6–7, lines 151–181
	8b	Rationalize the selection of test protocols, considering validity and reliability (e.g., coefficient of variation, familiarization)	Page 7, lines 166–169
	8c	Justify the smallest worthwhile effect or minimal clinically important difference	Page 7, lines 170–173 and 180–181
Randomization	9	Detail the exact mechanisms of generating and concealing the random allocation sequence	Page 4, lines 112–114
Blinding [b]	10	Document whether participants and/or researchers were aware of allocation (e.g., exit questionnaire)	Page 3, lines 90–91; Page 4 112–114<br>Page 6, lines 145–148
Standardization	11	Describe within- and between-participant controls (e.g., replication/reporting of diet, physical activity, sleep, and menstrual cycle)	Page 3, lines 87–89
Order Effects	12	Detail control of systematic influences of serial measurements (e.g., sequence effect in analysis model, wash-out interval)	Pages 3, lines 90–93

(continued)

PRESENT 2020 Checklist *(continued)*

Section	Item	Checklist	Page/Line Number (if applicable)
Statistics	13a	Specify the contrast for primary inferences (i.e., relative to the appropriate control, not changes from baseline in each group/condition)	Pages 9–10, lines 221–245<br>Tables 2–4
	13b	Clearly distinguish and fully justify any unplanned, interim, or exploratory subgroup analyses	N/A
	13c	Describe any adjustments for violated statistical assumptions and for relevant covariates (e.g., baseline measures)	N/A
**Results**			
	14a	Report the sample size at each phase from recruitment to analysis (with reasons for losses and exclusions)	Page 9, lines 208–217<br>Figure 1
	14b	Ensure data analysis matches research design, avoiding data pooling across groups/conditions (i.e., pseudoreplication)	Pages 9–10
	15a	Report SI units and report measures of central tendency, variability, and effect size/precision (confidence intervals)	Pages 9–10
	15b	Report individual data/responses (e.g., draw figures showing the raw data in each group/condition)	Figure 2
	15c	Document all relevant harms and unintended consequences observed	Page 9, lines 212–213
**Discussion**			
	16a	Present an objective and balanced interpretation of the observed data within the context of existing evidence	Pages 11–15
	16b	Consider the applicability and/or practical relevance of the research findings (e.g., external validity)	Page 15, lines 362–368
	16c	Acknowledge strengths and limitations of the research relevant to accurate interpretation (e.g., internal validity)	Page 15, lines 352–359
**Other**			
	17	State any relevant relationships (e.g., financial, technical, or material support)	Funding Sources Section
	18	Identify any publicly registered or published protocol (explaining any deviations)	N/A

[a] Adapted from the 2010 CONSORT (CONsolidated Standards Of Reporting Trials) checklist for reporting randomized controlled trials and can be used in conjunction with the associated paper that expands on each item.
[b] Items 7 (Interventions) and 10 (Blinding) are relevant for experimental research, including single-/double-blind contrasts of nutritional supplements.
